# PSMA-PET improves deep learning-based automated CT kidney segmentation

**DOI:** 10.1016/j.zemedi.2023.08.006

**Published:** 2023-09-02

**Authors:** Julian Leube, Matthias Horn, Philipp E. Hartrampf, Andreas K. Buck, Michael Lassmann, Johannes Tran-Gia

**Affiliations:** University Hospital Würzburg, Department of Nuclear Medicine, Oberdürrbacher Str. 6, 97080 Würzburg, Germany

**Keywords:** Kidney segmentation, PET/CT imaging, Automatic segmentation, Deep-learning based CT segmentation, Artificial intelligence

## Abstract

For dosimetry of radiopharmaceutical therapies, it is essential to determine the volume of relevant structures exposed to therapeutic radiation. For many radiopharmaceuticals, the kidneys represent an important organ-at-risk. To reduce the time required for kidney segmentation, which is often still performed manually, numerous approaches have been presented in recent years to apply deep learning-based methods for CT-based automated segmentation. While the automatic segmentation methods presented so far have been based solely on CT information, the aim of this work is to examine the added value of incorporating PSMA-PET data in the automatic kidney segmentation.

****Methods**:**

A total of 108 PET/CT examinations (53 [^68^Ga]Ga-PSMA-I&T and 55 [^18^F]F-PSMA-1007 examinations) were grouped to create a reference data set of manual segmentations of the kidney. These segmentations were performed by a human examiner. For each subject, two segmentations were carried out: one CT-based (detailed) segmentation and one PET-based (coarser) segmentation. Five different u-net based approaches were applied to the data set to perform an automated segmentation of the kidney: CT images only, PET images only (coarse segmentation), a combination of CT and PET images, a combination of CT images and a PET-based coarse mask, and a CT image, which had been pre-segmented using a PET-based coarse mask. A quantitative assessment of these approaches was performed based on a test data set of 20 patients, including Dice score, volume deviation and average Hausdorff distance between automated and manual segmentations. Additionally, a visual evaluation of automated segmentations for 100 additional (i.e., exclusively automatically segmented) patients was performed by a nuclear physician.

****Results**:**

Out of all approaches, the best results were achieved by using CT images which had been pre-segmented using a PET-based coarse mask as input. In addition, this method performed significantly better than the segmentation based solely on CT, which was supported by the visual examination of the additional segmentations. In 80% of the cases, the segmentations created by exploiting the PET-based pre-segmentation were preferred by the nuclear physician.

****Conclusion**:**

This study shows that deep-learning based kidney segmentation can be significantly improved through the addition of a PET-based pre-segmentation. The presented method was shown to be especially beneficial for kidneys with cysts or kidneys that are closely adjacent to other organs such as the spleen, liver or pancreas. In the future, this could lead to a considerable reduction in the time required for dosimetry calculations as well as an improvement in the results.

## 1 Introduction

Recent studies have demonstrated that a radiopharmaceutical therapy (RPT) with ^177^Lu-labelled prostate-specific membrane antigen (PSMA) is beneficial in treating castration-resistant prostate carcinoma [1], [2]. For optimal treatment, it is crucial to deliver the highest possible absorbed dose to the tumor during treatment, while preserving healthy tissue from toxicity. In contrast to many other typical organs at risk in RPT, the bladder, the submandibular and parotid glands, and the kidneys exhibit increased accumulation of PSMA and, consequently, are exposed to a higher absorbed dose [3] and are therefore considered a possible organ at risk that can result in treatment limitations. In consequence, PSMA-based RPTs involve the risk of nephrotoxicity [4], and it is advisable to estimate the renal absorbed dose across treatment cycles and the actual cumulated renal absorbed dose. Post-therapeutic ^177^Lu-PSMA SPECT/CT can be used for kidney dosimetry, which requires the location, size and shape of the kidneys. Although different morphological imaging modalities such as MRI could be used, these morphological parameters are usually determined from CT images, which are typically required for RPT planning. While manual segmentation can be the most accurate method if the operator is adequately trained and experienced, it has the major disadvantages of being time-consuming and that it can lead to significant inter- and intra-observer variability even for segmentation of the same kidney [5], [6], [7]. To mitigate these issues, numerous efforts have been proposed in recent years to perform segmentations using automated techniques such as deformable model-based [8], [9] or atlas-based [10] segmentations. With the recent success of machine learning, convolutional neural networks (CNNs) have been shown to hold great potential for CT-based renal segmentation [11], [12], [13].

Although a variety of CT-based automatic segmentations have been developed for the kidney, they are all based solely on morphological information. In this work, the idea is to enhance these morphology-based segmentations by including functional information from PSMA-PET. This promises to reduce the issues presented in [13] as cysts can readily be detected in PSMA-PET images due to a decrease in uptake. In addition, different activity concentrations in kidney, liver and spleen provide an additional means of differentiating these organs, which could in turn considerably improve automatic kidney segmentation based solely on CT.

In this study, a data set of 108 manual segmentations of PSMA-based PET/CT scans was used to train different CNNs to perform automatic kidney segmentation. Different approaches were applied to exploit the PET information in the automatic segmentation process. Finally, automated segmentations of 100 additional patients performed with and without the PET information were evaluated visually by an experienced nuclear physician.

## 2 Methods

### 2.1 Patient data available for our analysis

The convolutional neural network (CNN, see paragraph 2.4) was trained on 108 PSMA-based PET/CT patient scans acquired between 04/2019 and 11/2019 at the University Hospital Würzburg. All examinations had been performed with our standard-of-care protocol on a Siemens Biograph mCT 64 PET/CT scanner. Fifty-three patients had been examined with [^68^Ga]Ga-PSMA-I&T, and 55 with [^18^F]F-PSMA-1007. PET reconstruction had been performed using the built-in Siemens PSF reconstruction method (without time-of-flight) with 3 iterations and 24 subsets, with voxel size 4.07 × 4.07 × 5.00 mm^3^ and matrix size 200 × 200 × 171. Venous phase contrast-enhanced CT scans with resolutions between 0.64 × 0.64 × 5.00 mm^3^ and 0.98 × 0.98 × 5.00 mm^3^ (tube voltage 120 kV, in-plane matrix size: 512 × 512, pitch 1.4, mean CTDI_vol_ = 7.8 mGy) had been conducted for all analyzed patients as part of our standard-of-care. For a visual evaluation of the automatically generated segmentations, a second data set (ii) of 100 patients, examined at the University Hospital Würzburg between 04/2019 and 08/2019, was created. The imaging parameters were identical to the data set mentioned above and the same number of patients with [^68^Ga]Ga-PSMA-I&T based (50) and [^18^F]F-PSMA-1007 based PET examinations (50) were selected. Care was taken that no patient appeared in both data sets in order to avoid redundancies and data leakage. In addition, PET/CT scans of five additional patients, who had received only a native CT as opposed to a contrast-enhanced CT (resolution between 0.70 × 0.70 × 5.00 mm^3^ and 0.78 × 0.78 × 5.00 mm^3^, tube voltage 120 kV, in-plane matrix size: 512 × 512, pitch 1.4, mean CTDI_vol_ = 9.0 mGy, contrast agent *iomeprol®*), were used for the analysis of segmentation artifacts in case of incorrect input data. Given the retrospective character of this study, the local ethics committee waived the need for further approval for all data (waiver no. 20221201 01).

### 2.2 Manual segmentation process

Two kidney segmentations were performed by a human examiner for the 108 patients of the first data set (i) using the image computing platform 3D Slicer [14]: one based solely on the CT, and one based solely on the PET data. The CT-only segmentation was based on a visually chosen threshold (lower threshold between −12.5 HU and −0.3 HU, and upper threshold of up to 3071 HU), which was subsequently manually refined for each transversal slice, ensuring that both the cortex and the medulla were included, while excluding the renal pelvis, renal vessels, and potential kidney cysts as well as any other closely adjacent structures. The PET-only based segmentation was also initialized with a visually chosen threshold for each patient. For this segmentation, all voxels showing a PET signal in the area of the kidney were included. Morphological kidney structures (e.g., the renal pelvis) were not considered, but if necessary, nearby adjacent organs such as liver or spleen were excluded from the segmentation. This resulted in two segmentations available for each patient data set: a detailed CT-based segmentation, which is considered as ground truth, and a more coarse PET-based segmentation, which represents the accumulation of the tracer in the kidneys. The outcome of both segmentations was a binary matrix in which values of 1 or 0 indicate that a voxel does or does not belong to the kidney segmentation, respectively.

### 2.3 Data processing and data augmentation

The CT and the PET data sets as well as the two corresponding segmentations had to be adjusted as follows to ensure that both images had the same resolution and matrix size and enable training of a u-shaped convolutional neural network (u-net, [15]): first, the resolution of the CT and the associated segmentation (fine segmentation) was halved to save computation time and GPU memory requirements (in-plane matrix size: 512 × 512 → 256 × 256). To ensure a binary matrix for the segmentation, any interpolated values ≥0.5 and <0.5 were set to 1 and 0, respectively. Subsequently, the PET and its segmentation (coarse segmentation) were linearly interpolated to reach the same matrix size (in-plane matrix size: 200 × 200 → 256 × 256). Finally, an array of 128 slices, centered on the abdominal region containing the kidneys, was cut out of all resulting 3D matrices, resulting in a matrix size of 256 × 256 × 128. All CT and PET scans were individually scaled to an interval of [0,1] by normalization to the maximum voxel value of the respective volume. When designing the data processing pipeline, a network was trained for testing purposes, where the CT was clipped to a maximum value of 1000 HU before normalization. As this did not result in any difference in performance, it was decided not to clip the CT values prior to normalization.

The total of 108 data sets was divided into training (78 patients), validation (10 patients), and test data (20 patients), taking care to balance the number of ^18^F-based and ^68^Ga-based PSMA-PET data sets in each subset. To reduce the effect of overfitting, data augmentation was performed for the training and validation data sets in a similar manner as described in [13] using the following techniques: random zooming of a factor between 0.8 and 1.2, random rotation around the longitudinal axis (angle between −7.5° and 7.5°), random voxel shift along all axes (shift between 1 and 5 voxels) and random flipping of the whole volume axially and sagitally. For the CT, an additional random Hounsfield Unit shift between −7.5 and 7.5 HU was applied. For each patient, 9 different augmented versions were generated, increasing the total number of training and validation data sets to 780 and 100, respectively.

### 2.4 Network architecture and selection of hyperparameters

**Figure 1:**
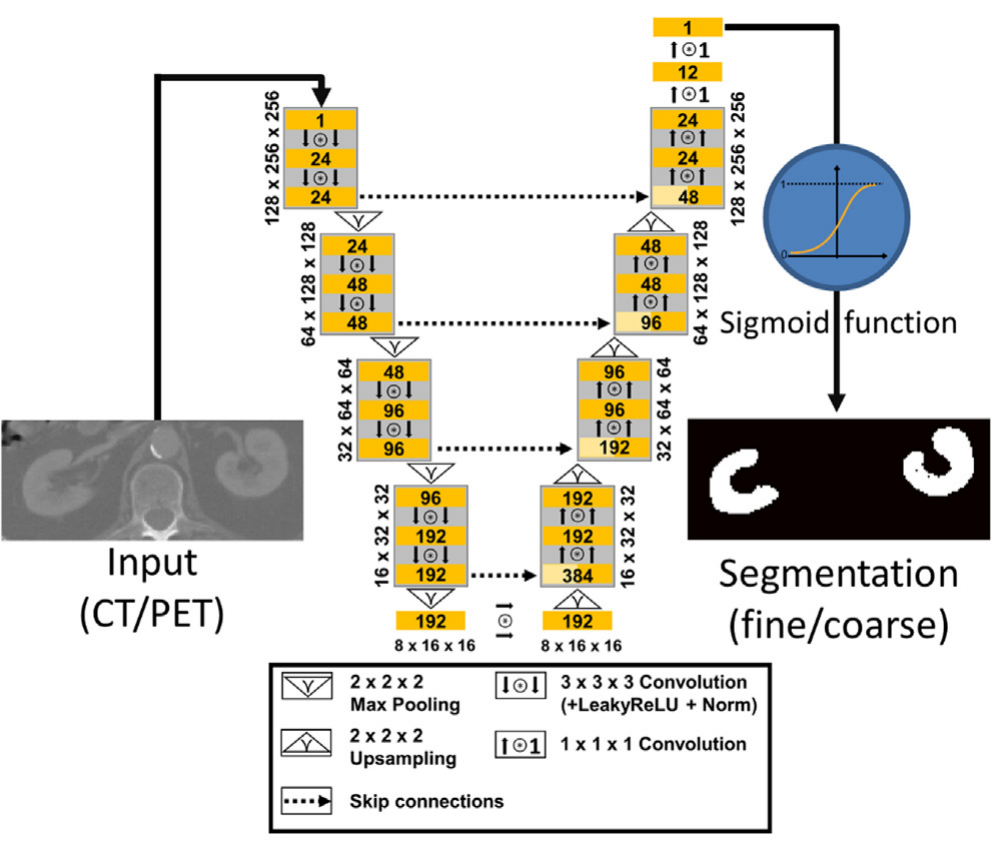
Architecture of the u-net used in this study.

All u-nets developed in this study are based on the fastMRI architecture [16], and they were implemented using the PyTorch library [17]. A representation of the architecture is given in Fig. 1. All two-dimensional operations were exchanged with the respective three-dimensional counterpart. The number of downsampling layers was 4 and the number of output channels after the first convolution was set to 24. After each convolution block, an Instance Normalization was performed and a Leaky Rectified Linear Unit activation function (Leaky ReLU, negative slope of 0.2) was applied. At the end of the decoder path, a sigmoid function was applied to keep the output values in an interval of [0,1]. Training was performed without early stop rules for 60 epochs based on the Adam optimizer [18]. The initial learning rate was set to $7\times 10^{-5}$ and was halved every 20 epochs. Dropout with a probability of 0.1 was applied to all convolutions, to reduce the effect of overfitting. Dice-BCE-Loss, a combination of Dice Score [19] and binary cross-entropy was chosen as loss function:

(1) $$L\left(x,y\right)=\left(1-\frac{2\sum_{i=1}^{N}x_{i}y_{i}}{\sum_{i=1}^{N}x_{i}+\sum_{i=1}^{N}y_{i}}\right)-\frac{1}{N}\left(\sum_{i=1}^{N}y_{i}\log x_{i}+\left(1-y_{i}\right)\log\left(1-x_{i}\right)\right)$$

Here, x is the prediction by the u-net, y is the true segmentation, and N is the total number of voxels (256 × 256 × 128). Validation loss was calculated after every epoch, saving the parameters of the network only if the validation loss reached a new minimum to avoid overfitting.

### 2.5 Comparison of different approaches for kidney segmentation

**Figure 2:**
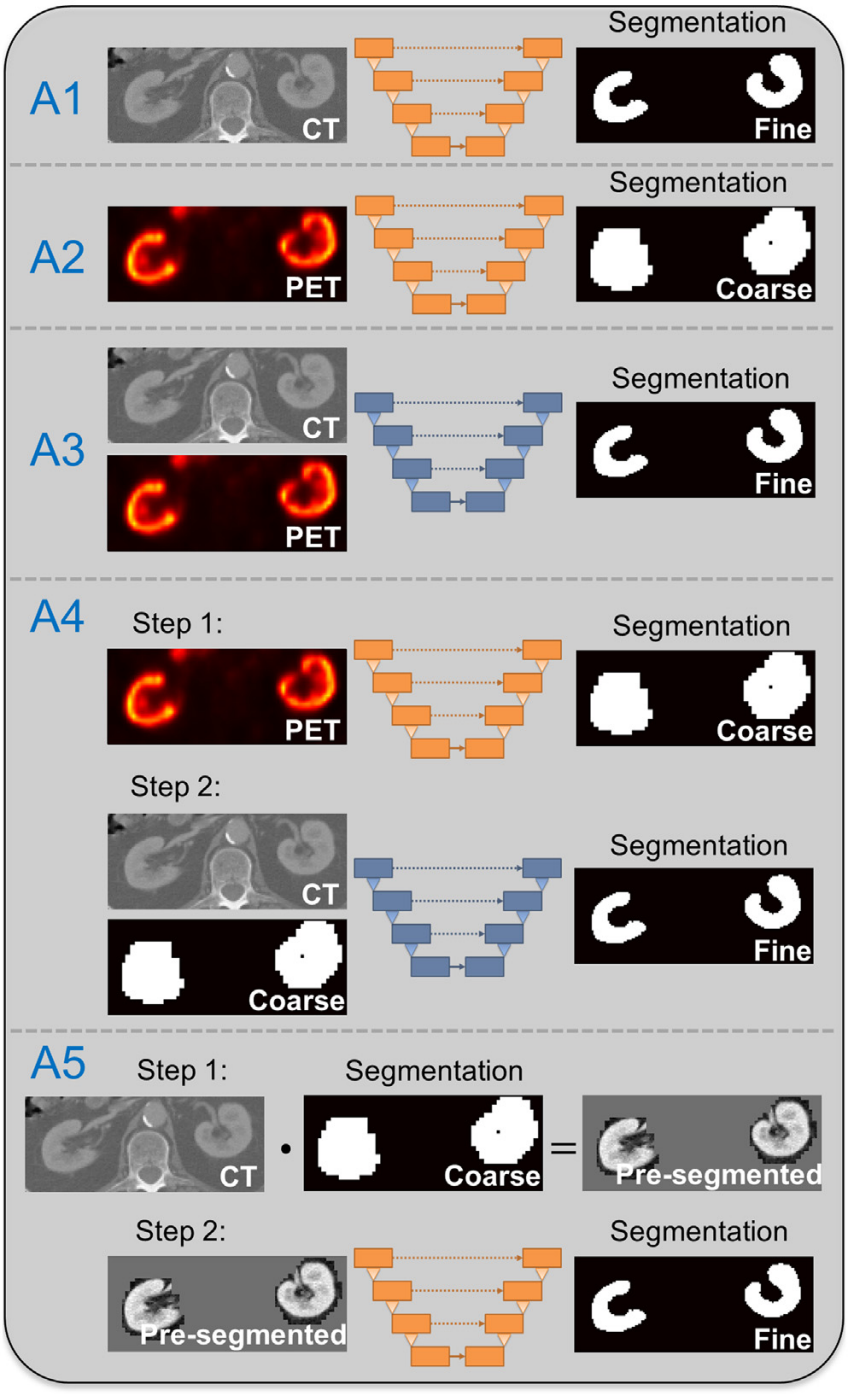
Schematic overview of u-net based kidney segmentation approaches applied in this work. While approaches A1 and A2 perform simple end-to-end training from image to segmentation, approaches A3 to A5 exploit information from both imaging modalities. The u-nets for approach A3 and A4 (step 2) use a two-channel input (orange u-net: one-channel input, blue u-net: two-channel input).

To assess how the performance of a u-net based segmentation trained only based on CT data can be improved by the addition of PET information, a total of five different approaches A1 to A5 were implemented, which are summarized in Table 1 and Fig. 2. While approaches A1 and A2 perform simple end-to-end training from image to segmentation, approaches A3 to A5 exploit information from both imaging modalities. Since the PET segmentation of A2 results in a very coarse segmentation in contrast to the fine segmentations of all other approaches involving CT images, A2 is not considered in the final evaluation. Approaches A4 and A5 consist of two steps: first, a pre-segmentation based on the PET data is performed using A2; then, the information from the coarse pre-segmentation is exploited in different ways to generate better input for a more accurate fine segmentation. Therefore, unlike the other three approaches (A1 to A3), A4 and A5 are not trained end-to end. For approach A5 the PET-based mask was enlarged by two voxels along each axis before the voxelwise multiplication, to prevent parts of the kidney from being cut off by the pre-segmentation.

**Table 1:** Summary of applied segmentation approaches A1 to A5.

	**Input**	**Output**
A1	CT image	Fine segmentation
A2	PET image	Coarse segmentation
A3	2 channels: CT image + PET image	Fine segmentation
A4	Step 1 (A2): PET image	Step 1 (A2): Coarse segmentation
	Step 2 (2 channels): CT image + Coarse segmentation	Step 2: Fine segmentation
A5	Step 1 (A2): PET image	Step 1 (A2): Coarse segmentation
	Step 2 (single channel): CT image pre-segmented based on coarse segmentation	Step 2: Fine segmentation

### 2.6 Evaluation metrics

A set of metrics was implemented to evaluate the performance of the different deep learning-based fine segmentation methods (A1, A3, A4 and A5) when compared to the ground truth (i.e., the manual CT segmentation) by applying them to all patient scans in the test data set. The Dice score [19] was chosen as an overlap-based metric, while the average Hausdorff distance (AHD) was chosen as a distance-based metric [20]. The Dice score (DSC), which is also used in the loss function, describes the level of overlap of two segmentations x and y:

(2) $$DSC\left(x,y\right)=\left(\frac{2\sum_{i=1}^{N}x_{i}y_{i}}{\sum_{i=1}^{N}x_{i}+\sum_{i=1}^{N}y_{i}}\right)$$

While a DSC of 1 corresponds to a perfect overlap between both segmentations, a DSC of 0 means no overlap.

Given two point sets A and B, corresponding to the segmentations x and y, the AHD describes the average distance from a point in one set A or B to the closet point in the other set:

(3) $$AHD\left(A,B\right)=\left(\frac{1}{N}\sum_{a\in A}\min_{b\in B}{\left\|a-b\right\|}_{2}+\frac{1}{M}\sum_{b\in B}\min_{a\in A}{\left\|b-a\right\|}_{2}\right)/2$$

Here, M and N are defined as the number of points of the set A and B, respectively, and ${\left\|∙\right\|}_{2}$ is the L2-norm.

In addition, the volumes of the manually and the automatically segmented kidneys were determined and their volume deviation (VD) was calculated as:

(4) $$VD\left(x,y\right)=\frac{\left|\sum_{i=1}^{N}x_{i}-\sum_{i=1}^{N}y_{i}\right|}{\sum_{i=1}^{N}y_{i}}∙100\%$$

Two-sided Wilcoxon tests were used to investigate differences in the evaluation metrics between the segmentation methods.

For approach A5, two-sided Mann Whitney U tests were applied to investigate whether the radiopharmaceutical used for the PSMA-PET acquisition had a significant impact on the performance of the segmentation. In the test data set, there were 9 patients examined with [^68^Ga]Ga-PSMA-I&T and 11 patients examined with [^18^F]F-PSMA-1007.

### 2.7 Visual assessment of automatic segmentations for which no manual segmentation was available

**Figure 3:**
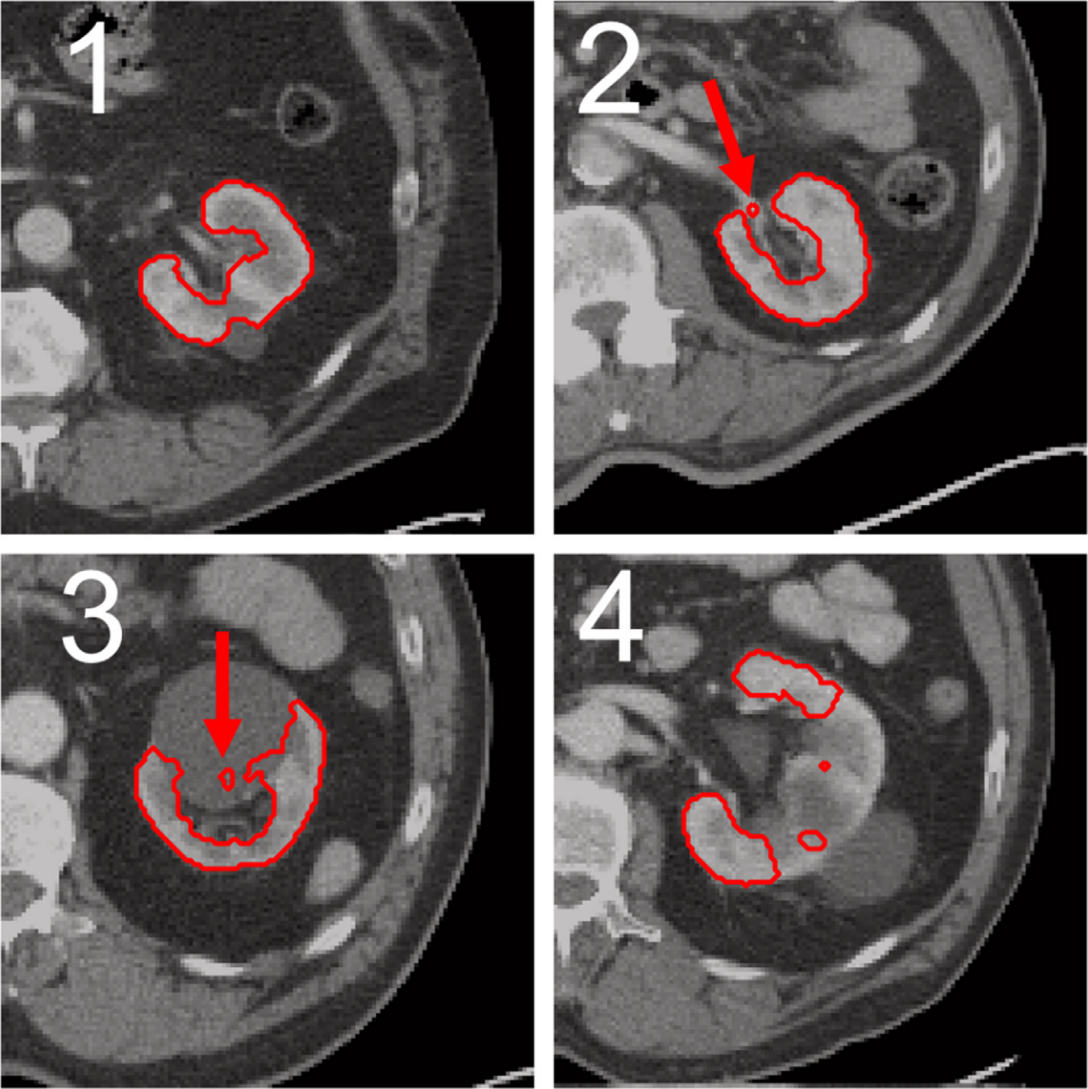
Exemplary representation of automatically generated segmentation of the four scoring values. All segmentations were created solely based on CT using approach A1. For the segmentation, which was rated with a score of 1 (upper left, image 1), no errors are recognizable. For the kidney on the top right (image 2) a small part of the renal hilus was segmented incorrectly (indicated by the red arrow), resulting in a score of 2. Bottom left (image 3) a segmentation containing parts of a cyst is shown (indicated by red arrow), resulting in a score of 3. For the segmentation (lower right, image 4), which was rated with a score of 4, rough errors are visible, as large parts of the kidney are not segmented.

For further performance testing, the solely CT-based approach (A1) and the hybrid approach (A5) identified as most effective in the performance analysis were applied for automated segmentation of 100 additional “unseen” patient data sets (i.e., no manual segmentation was available and the data were not part of the quantitative analysis) of data set (ii). Both segmentations were blindly graded by an experienced nuclear physician using a score that ranged from 1 (nearly perfect) to 4 (rough errors in the segmentation). More details on the score are provided in Table 2. A visual example of each of these scores can be found in Fig. 3. In addition to awarding the score, the nuclear physician also selected the best overall segmentation of both kidneys for each patient.

**Table 2:** Classification of the scoring values for the visual evaluation of the segmentations.

**Score**	**Match**	**Description**
1	95–100%	Nearly perfect
2	90–95%	Small errors
3	85–90%	Clear errors
4	80–85%	Rough errors

## 3 Results

### 3.1 Quantitative analysis of the test data set

Table 3 lists mean DSC, volume deviation, and AHD between automated (A1, A3, A4, and A5) and manual segmentations for all 20 patients in the test data set. The highest DSC (left kidney: 0.937, right kidney: 0.935) and the lowest AHD value (0.058 mm for both kidneys) were found for A5 (as described in the Methods section, A2 is not part of this comparison as it does only provide a coarse segmentation). In addition, the performance of A5 proved to be significantly (two-sided Wilcoxon test, p < 0.05) better than the performance of A1, A3 and A4 based on DSC and AHD. The better performance of A5 also becomes evident from the violin plots in Fig. 5 by a larger/smaller/smaller median DSC/volume deviation/AHD (red violins), respectively. However, no statistically significant difference in the volume deviations was found between all four approaches.

**Table 3:** Mean Dice score (DSC), volume deviation and average Hausdorff distance (AHD) between manual and automatic segmentations. Standard deviations are given in parentheses. The best scores for all metrics are written bold (except PET segmentation A2). Significant differences between approaches A1, A3 and A4 and approach A5 were marked for all three metrics (*).

**Abbreviation**	**Left kidney**			**Right kidney**		
	**DSC**	**VD**	**AHD [mm]**	**DSC**	**VD**	**AHD [mm]**
A1	0.924* (0.019)	7.14% (5.29%)	0.077* (0.029)	0.924* (0.271)	8.03% (7.23%)	0.072* (0.039)
A2	0.935 (0.032)	8.69% (7.04%)	0.121 (0.132)	0.938 (0.026)	7.86% (6.07%)	0.133 (0.139)
A3	0.928* (0.013)	6.26% (3.98%)	0.068* (0.019)	0.929* (0.019)	6.80% (5.26%)	0.068* (0.026)
A4	0.926* (0.020)	7.32% (7.52%)	0.073* (0.031)	0.922* (0.032)	8.87% (9.58%)	0.076* (0.042)
A5	**0.937** (0.014)	**5.53%** (4.35%)	**0.058** (0.018)	**0.935** (0.018)	**6.74%** (6.15%)	**0.058** (0.020)

**Figure 4:**
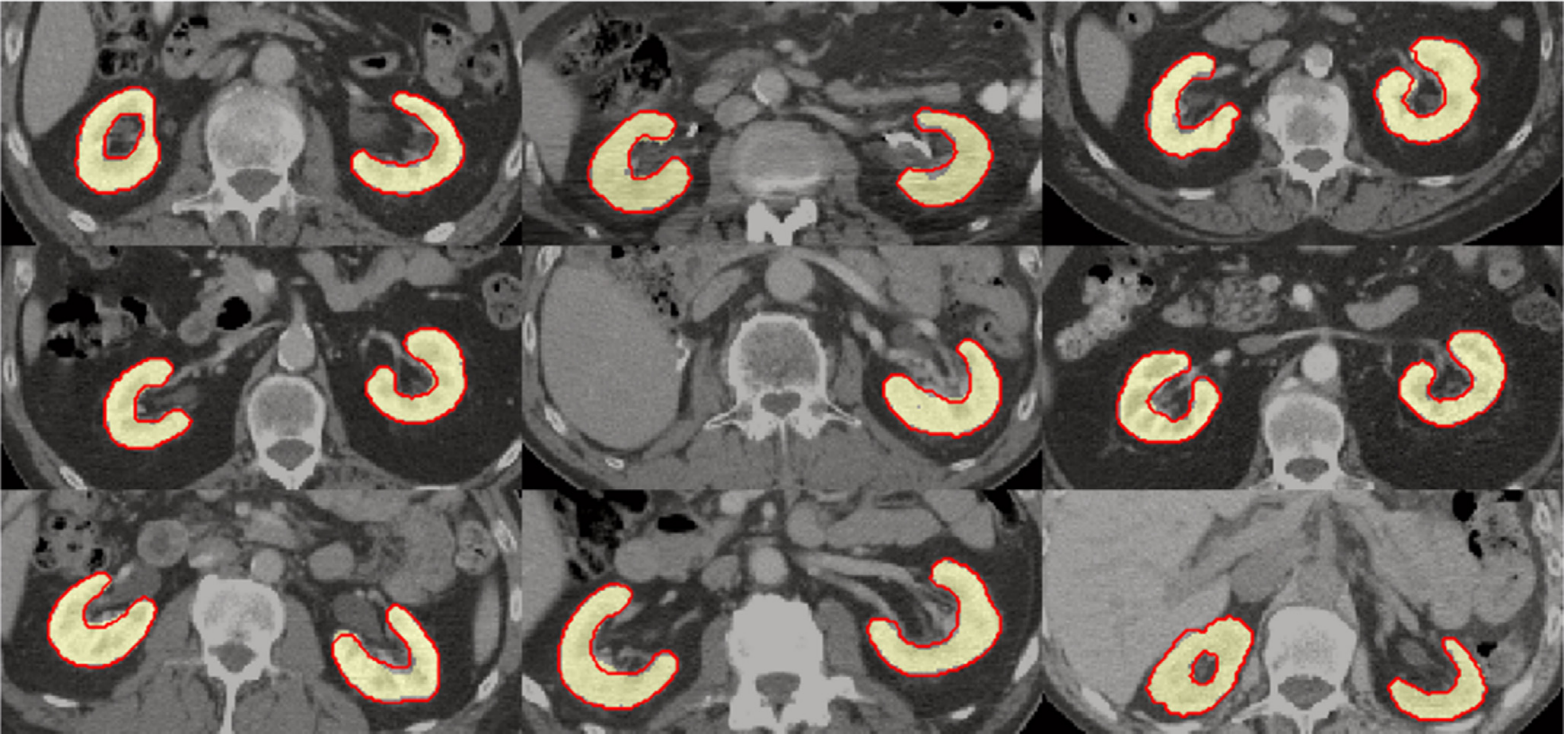
Example segmentations for nine patients of the test data set. Yellow area: manual segmentation. Red contour: automated segmentation created by approach A5.

Fig. 4 shows example axial CT slices of the kidneys for nine patients of the test data set, as well as the corresponding manual and automated segmentation using A5.

**Figure 5:**
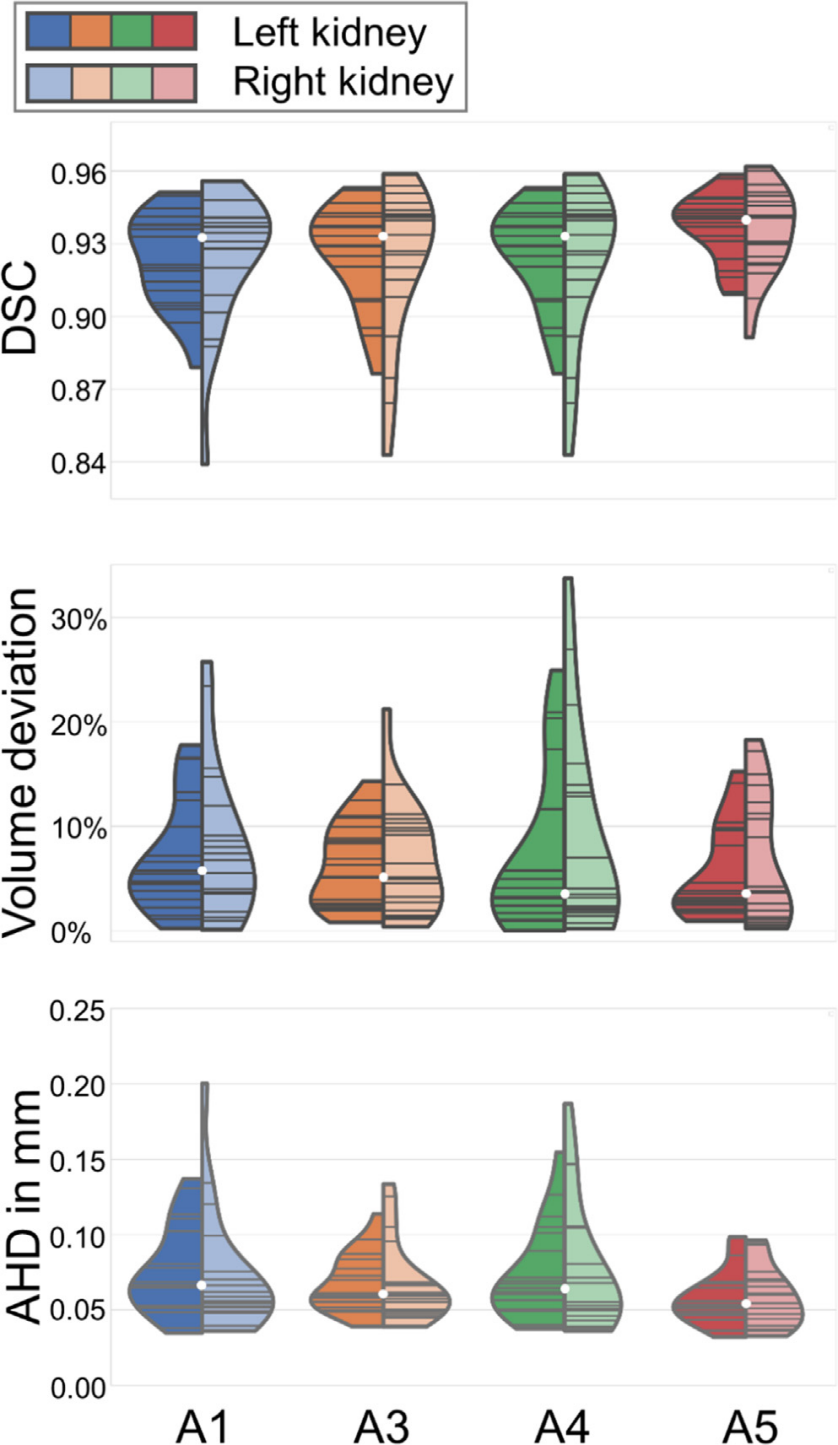
Violin plots illustrating the performance of approaches A1, A3, A4, and A5 for all evaluation metrics (top to bottom: Dice score (DSC), volume deviation, and (AHD) average Hausdorff distance) separately for left and right kidney (left and right part of each violin). The white dot represents the median and the gray lines illustrate the data points. The segmentation method is better the closer the DSC value is to 1 or the VD and AHD values are to zero.

According to a two-sided Wilcoxon test, no systematic differences (p > 0.05) were found between left and right kidney segmentations for all evaluation metrics and approaches.

No radiopharmaceutical-dependent difference in DSC, and thus performance of A5, was found for the left (p = 0.96) and the right (p = 0.43) kidney.

### 3.2 Analysis of the visual evaluation

Table 4 shows the relative frequencies of the visual scores assigned by the nuclear physician for segmentations based on A1 and A5. For A5, score 1 was awarded most often by a significant margin (71%/79.8% for left/right kidney), while for A1, scores 1 and 2 were awarded almost equally often (score 1: 42%/58.6%; score 2: 46%/30.3% for left/right kidney). The mean score shows the superior performance of approach A5 (1.37/1.22) in comparison to A1 (1.76/1.54), which is underlined by a significant difference based on a paired Wilcoxon test (p < 0.01). Regarding the influence of the radiopharmaceutical (i.e., the combination of radionuclide and PSMA ligand), the choice between [^68^Ga]Ga-PSMA-I&T and [^18^F]F-PSMA-1007 had no significant impact on the segmentations using A5 according to the visual evaluation (Mann-Whitney U test, p = 0.29/0.96 for left/right kidney).

**Table 4:** Absolute frequency of visual scores assigned to the 100 non-manually segmented automatic segmentations.

**Score**	**Absolute frequency, %**			
	**Left kidney**		**Right kidney**	
	**A1**	**A5**	**A1**	**A5**
1	42.0	71.0	58.6	79.8
2	46.0	23.0	30.3	17.2
3	6.0	4.0	8.1	3.0
4	6.0	2.0	3.0	0.0

## 4 Discussion

### 4.1 Comparison of Dice scores with the literature

From all approaches applied in our study, the pre-segmentation method A5 (CT image pre-segmented using a PET-based coarse mask as input) resulted in the best outcomes regarding Dice score and Average Hausdorff distance. In a recent review article, Pandey and Gupta compared different approaches and algorithms for automated kidney segmentation in abdominal images [21]: segmentation based on mathematical models (e.g. deformable model-based segmentation [8], [9]) and algorithms (e.g. fast grow-cut algorithm [22], atlas-based segmentations [10] and machine learning algorithms). The latter are divided into classification algorithms, such as random forest [23], [24] or deep learning methods using convolutional neural networks (CNN) [11], [12], [13]. Their analysis revealed that the best results can be achieved using deep learning methods. For instance, Türk et al. [11] trained a hybrid V-net for kidney segmentation based on contrast-enhanced CT images (210 patients of the KiTS19 data set) and obtained a DSC of 0.977 (difference to the DSCs of our approach A5: 0.041). Similarly, Lin et al. [12] used a cascaded 3D u-net structure (data set: CT urograms of 441 patients) and reported a comparable DSC of 0.973 (difference to DSCs of our approach A5: 0.037). The authors used a two-stage approach, referred to as *coarse-to-fine*: After a coarse segmentation of the kidneys using a first CNN, they are more accurately segmented using a second CNN. The training in this study was therefore not end-to-end, just as for our approaches A4 and A5. Both methods are solely based on CT and achieved noticeably higher DSCs than the DSCs presented in our study. This could be explained by more advanced neural network architectures applied in those studies ([11]: hybrid V-net structure, [12]: cascaded 3D u-net structure) as opposed to the unmodified 3D u-net structure (fastMRI [16]) used in our work. However, Gut et al. [25] recently presented a comparison between the basic u-net and five major extensions (ResUNet [26], UNet++ [27], UNet 3+ [28], CS2-Net [29], and CPFNet [30]) to disentangle the influence of model architecture, model training, and parameter settings on their performance for segmentation of medical image data. Their study demonstrated that the architecture variants do not improve the quality of inference related to the basic u-net architecture while resource demands increase. The primary goal of this work was to incorporate PET information into the typically CT-based segmentation and only secondarily to maximize the performance of the segmentation. Therefore, we decided to keep the architecture as simple as possible and focus on the basic u-net.

**Figure 6:**
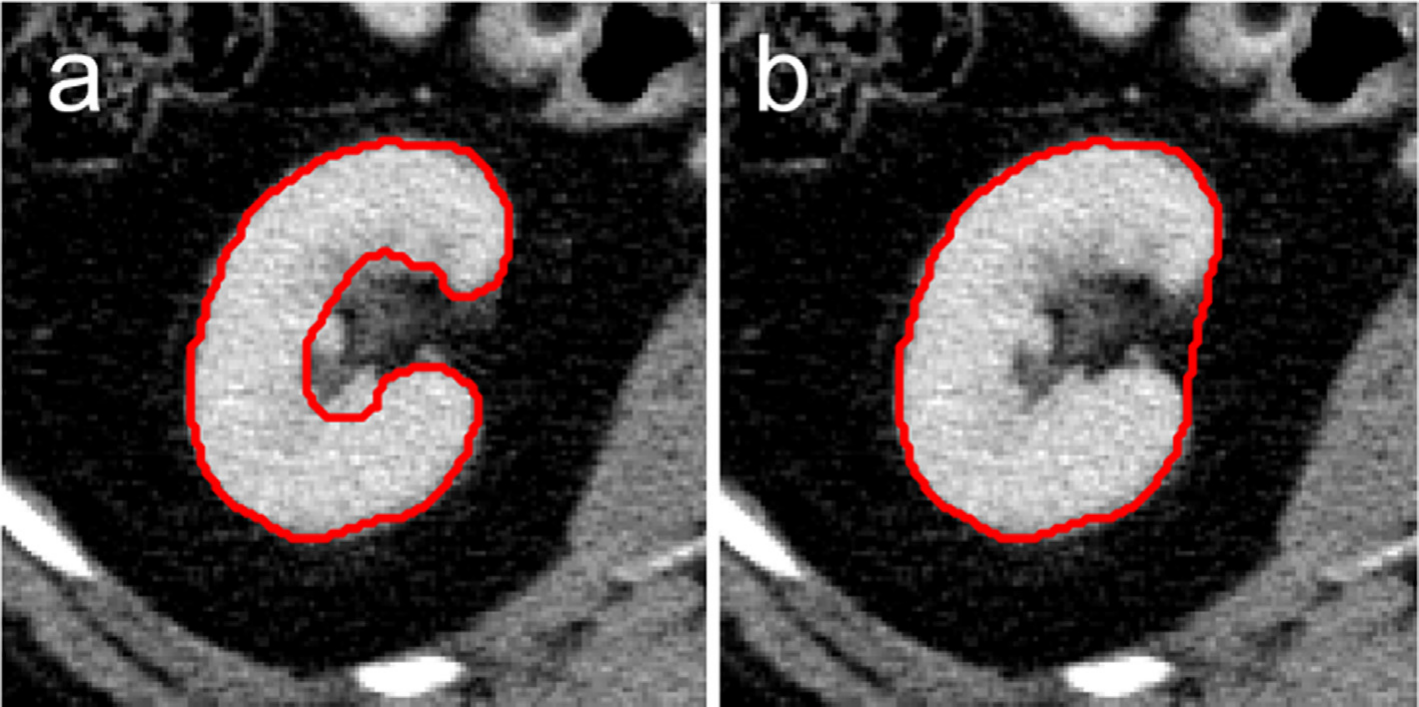
Illustration of different levels of accuracy of renal segmentations. a: more detailed segmentations as performed in this study, excluding the renal pelvis and vessels. b: coarser segmentation as performed in [11], [12].

Another possible reason for the difference in DSC ranges could be the quality and accuracy of the manual segmentations, which is illustrated in Fig. 6. In our study, care was taken to include the renal cortex and medulla, while excluding pelvis and vessels. In contrast, a closer look at the manual segmentations in [11], [12] reveals that the renal vessels were included in these segmentations (Fig. 6). This results in a visual appearance of an ellipsoid that appears much less complex than the actual bean-like kidney shape. This makes segmentation easier for both human users and u-nets, which in turn translates into higher DSC values.

In the publication by Jackson et al. [13], it was shown that deep learning-based automatic segmentation of the kidney can be used for ^177^Lu SPECT/CT based dosimetry of RPT (low-dose native CT images of 89 patients). The study achieved lower DSCs (0.86/0.91 for left/right kidney, difference to the DSCs of our approach A5: −0.077/−0.025) than all segmentation techniques presented in this work –both with and without PET information– and the methods presented in the two studies [11], [12] mentioned above. This may be explained by the use of full-dose contrast-enhanced CT images in this work as well as in [11], [12], as opposed to low-dose native CT images that were used in [13]. The contrast enhancement facilitates a visual differentiation between the kidneys and surrounding organs.

### 4.2 Benefits of the pre-segmentation approach

**Figure 7:**
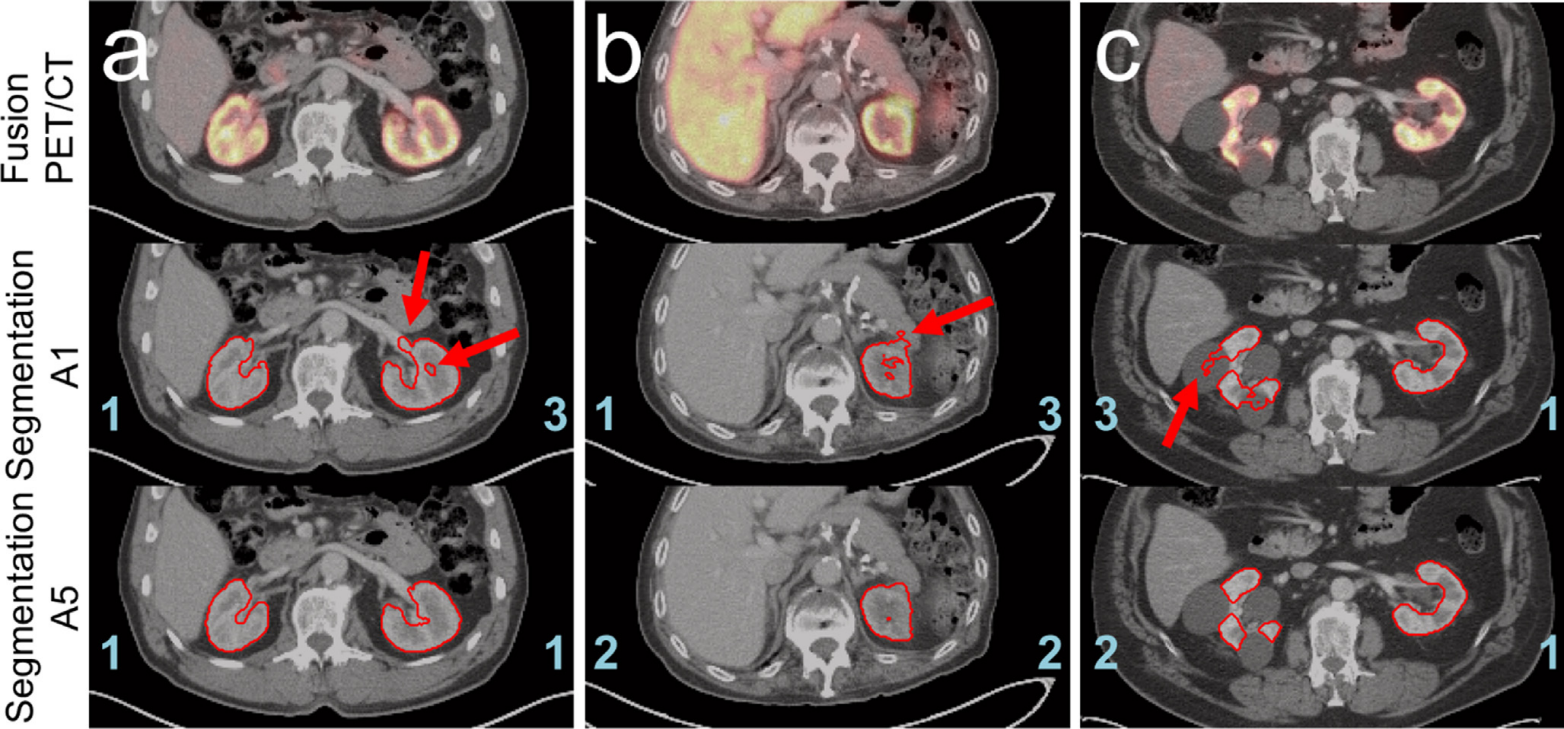
Examples highlighting the benefits of the pre-segmentation approach A5. PET/CT fusion (top), CT with the contour of the segmentation created by A1 (middle), and CT with contour of the segmentation created by A5 (bottom) are depicted for three different patients from the visual evaluation data. The assigned scores for the segmentation are indicated in blue and the mentioned artifacts are highlighted by red arrows: a: too much of the left renal hilus is segmented by approach A1. In addition, a hole in the left kidney marrow is visible. The segmentation using approach A5, on the other hand, does not show these inadequacies. b: both segmentations recognize that the right kidney is missing. However, part of the pancreas is included in the A1 segmentation. This artifact does not occur for the pre-segmentation method A5. c: patient with large cysts in the right kidney. Approach A1 exhibits major problems in distinguishing between renal tissue and cysts, thus large sections of the cysts are misclassified. In contrast, the pre-segmentation approach A5 is significantly more effective at segmenting the kidney tissue excluding the cysts.

Through the visual evaluation of the 100 additional automatic segmentations, it was possible to assess, in which cases PET pre-segmentation was advantageous. Representative examples are given in Fig. 7. It is evident that the pre-segmentation method yields better results for kidneys with cysts or kidneys that are close to adjacent organs such as the pancreas or liver.

When considering dosimetry based on the Medical Internal Radiation Dose (MIRD) schema [31], erroneous kidney segmentations can lead to errors in dose calculations in two ways: i) The absorbed dose is inversely proportional to the target organ mass. Since organ masses are typically determined based on volume, errors in kidney segmentation automatically result in incorrect organ masses, which in turn result incorrect absorbed doses. ii) The absorbed dose is dependent on the time-integrated activity in the source organ. In SPECT/CT-based activity determination, activity is typically determined using CT-based VOIs, which are transferred to the SPECT-based activity distribution to determine source organ activity (organ-based dosimetry), or to perform Monte Carlo radiation transport simulations (voxel-based dosimetry [32]). For these reasons, an improved spatial differentiation of the active volume within the segmentation process, in most cases, also leads to a more accurate dosimetry.

One must also be aware that approach A5 could leave out parts of the kidney that are not in the pre-segmentation mask. Therefore, we enlarged the pre-segmentation mask by 2 voxels along each axis before multiplication with the CT to ensure not to leave out any important structures of the kidneys.

### 4.3 Artifacts and pitfalls

Various studies have shown that the performance of neural networks can be strongly affected by disturbances in the input data [33], [34]. A number of cases were specifically tested and identified in this study for which artifacts and problems might occur when incorrect or deviating input data is used, a subject referred to as domain adaptation in the machine learning community.

Furthermore, the effect of using native CT instead of contrast-enhanced CT as input was tested for five different patients, from whom only native CT images were available. The doses of the contrast enhanced CTs and the native CTs were in a comparable range (contrast-enhanced CT: mean CTDI_vol_ = 7.8 mGy, native CT: mean CTDI_vol_ = 9.0 mGy). As expected, several segmentation issues were discovered for approach A1: cysts as well as neighboring organs, such as the spleen and liver, were partially included in the kidney segmentation; in addition, the u-net had problems in determining the exact boundaries of the kidney. Encouragingly, the pre-segmentation approach A5 showed no signs of such problems and was able to successfully segment the kidneys. Based on these observations, automatic segmentation based on native CTs could probably benefit most from pre-segmentation using PET. However, further analysis on the basis of a corresponding data set will be needed to support this assumption. In addition, it should be mentioned that the networks were not retrained (e.g., using transfer learning methods), which is expected to considerably improve their performance with little training data required.

## 5 Conclusion

The majority of deep learning methods for kidney segmentation are solely based on the anatomical information from CT. Based on 108 manual kidney segmentations of PSMA-PET/CT patient data, we demonstrated that the combination of PET and CT information, which involves two sequentially applied neural networks, provides better results than a purely CT based segmentation. On a test data set of 20 patients, a PET-based pre-segmentation of the CT input significantly improved the results of other deep learning-based segmentations (e.g., solely based on CT) with regard to two commonly used evaluation metrics (Dice score and average Hausdorff distance). The superior performance of this method was confirmed by an experienced nuclear physician’s visual evaluation of an additional 100 automatically segmented patient data sets.

## Author contributions

All authors contributed to the study conception and design. MH performed the manual segmentations of the kidneys. PH performed the scoring of the automatic segmentations. JL and JTG developed the methodology, trained the u-nets, performed the data interpretation and analysis, and prepared the first draft of the manuscript. All authors commented on previous versions of the manuscript. All authors read and approved the final manuscript.

## Funding

This study was funded by a grant of the German Research Foundation (Deutsche Forschungsgemeinschaft TR1380/1-1) and partially by a grant from the German Federal Ministry of Education and Research (13GW0357B). The funders had no role in the design of the study; in the collection, analyses, or interpretation of data; in the writing of the manuscript, or in the decision to publish the results.

## Declaration of Competing Interest

The authors declare the following financial interests/personal relationships which may be considered as potential competing interests: Michael Lassmann has received institutional grants by IPSEN Pharma, Nordic Nanovector, and Novartis. No other potential conflicts of interest relevant to this article exist.
